# Notes and News

**Published:** 1892-07-02

**Authors:** 


### Notes and News.

Collected and Communicated.

"The Million," a new paper, partly illustrated by a new process in water-colour printing, has increased its pages to twelve, all of which are supplied to the public for one penny.

Amongst the interesting events of the past week may be included the festival dinner of the West London Hospital, at which Sir Algernon Borthwick presided, and which has resulted in contributions of £2,619 being added to the funds of the institution; the garden party at the Cancer Hospital, Brompton, at which a large and distinguished party were present; the meeting in connection with Dr. Barnardo's Homes; and the Lady Mayoress' rose fête.

A very interesting ceremony took place on Saturday in the opening of the New Beach Rocks Convalescent Home at Sandgate, in connection with the London Samaritan Society and Homerton Mission, by Sir Edward and Lady Watkin. The building, which is immediately under the Shorncliffe heights, and thus sheltered on the north and east, has accommodation for 250 inmates. It faces the sea, and leads by steps to the beach. It consists of a central block and two wings, and wide balconies running round it for an excellent promenade for patients in wet weather. It is interesting to note that all the beds have been separately presented and bear the donor's name. The whole establishment was erected at a cost of £20,000.

At the opening of the new clinical theatre at the National Hospital for Paralysed and Epileptic, Queen's square, the Earl of Strafford opened the proceedings, and Sir James Paget delivered the inaugural address. Sir James, in his speech, paid a just tribute to the merits of the hospital. He said that "he knew no hospital which had more perfectly carried out the purpose for which it was established, for whilst the institution had been an admirable one of its kind for the healing of disease, there had been at the same time a constant carrying on of research and teaching, and no year had passed since the foundation of the institution which had not been marked by some distinct and definite increase in the knowledge of medicine and surgery." He further referred to the great improvements in the nursing arrangements of the hospital.

It is interesting to learn that M. Pasteur acknowledges that he received inspiration in his studies on hydrophobia from Madame Greville's description of the Muscovite treatment of that dreadful disease.

It must be a matter of regret to all concerned in St. Bartholomew's Hospital that Sir Sidney Waterlow is about to resign the office of Treasurer to the Hospital. Sir Sidney has rendered eighteen years' good service. He will probably be succeeded by Sir Trevor Lawrence, M.P.

The 26th anniversary of Dr. Barnardo's Homes, held on 22nd ult. in the Royal Albert Hall, under the presidency of Lord Kinnaird, was a most important occasion. The Royal boxes were placed at Dr. Barnardo's disposal, and no less a sum than £2,400 was contributed towards the work. The choir on the occasion consisted of about 1,500 children, with a smaller number of adult voices. They were supported by an excellent brass band belonging to the homes. Practical illustrations of most of the fifteen different trades taught in the homes were given during the evening, and some of the boys were put through naval and military exercises in a most creditable manner, and were much applauded by the audience. The fact that some 4,400 boys and girls, the "residuum" of society, who would have filled our workhouses, our infirmaries, and our jails are being disciplined, and trained, and taught, improved physically, mentally, and morally reflects to the enormous energy of Dr. Barnardo and to his great power of organisation. The income for the past year was £131,000, being an increase of £21,000 on the year 1890. There are now forty-eight different homes or branches.

A Dutch medical man, Dr. Voûte, of Amsterdam, has just published his impressions of the various institutions for medical aid to children throughout the principal countries of the Continent after a personal visit to most of them. The French criticism of his work, so far as French hospitals is concerned, is that it is not sufficiently thorough. Nevertheless, Dr. Voûte has evidently taken the pains to visit a considerable number of establishments, and to have made himself personally acquainted with the directors, his remarks on an institution being in most cases accompanied by a short character sketch of the director, for which we can hardly imagine they can have been quite prepared. Whilst individual hospitals seem to have met with his approval, Dr. Voûte remarks that the French hospital service suffers in comparison with that of some other countries, as the medical officers are too migratory, and the study of special subjects is therefore not so thorough. In respect to medical treatment, he complains of the abuse of blistering agents, emetics, cupping instruments, and that infantile therapeutics in Paris call for reform. On the other hand, he declares the preparation of iron, arsenic, and of antipyrine to be preferable to those employed in Holland or Germany. With the establishments of Berlin, Dr. Voûte appears to be very satisfied, and he admires the hygienic arrangements, simplicity of treatment, and judicious use of drugs. The treatment employed in diphtheria does not, however, meet with his approval. Vienna comes in for scant praise, and Buda-Pest and Berne, with examples of medical and surgical treatment proceeding from the same medical officer, are of course held up for reform.

